# Homelessness and Organ Donor–Derived *Bartonella quintana* Infection

**DOI:** 10.3201/eid3012.240389

**Published:** 2024-12

**Authors:** Rachel Henderson, Emily Mosites, Jane E. Koehler, Carl Boodman, Grace E. Marx

**Affiliations:** University of Colorado School of Medicine, Fort Collins, Colorado, USA (R. Henderson); Multnomah County Health Department, Portland, Oregon, USA (E. Mosites); University of California, San Francisco, California, USA (J.E. Koehler); University of Manitoba, Winnipeg, Manitoba, Canada (C. Boodman); Institute of Tropical Medicine, Antwerp, Belgium (C. Boodman); Centers for Disease Control and Prevention, Fort Collins, Colorado, USA (G.E. Marx)

**Keywords:** Bartonella quintana, bartonellosis, bacteria, louse, lice, homeless, homelessness, Pediculus humanus humanus, parasites, pediculosis, United States

## Abstract

Louseborne *Bartonella quintana* infections in the United States occur almost exclusively among persons experiencing homelessness because of inadequate access to hygiene resources. Homelessness is increasing, and persons experiencing homelessness can be organ donors, despite barriers to receiving donated organs themselves. Recent reports have documented *B. quintana* transmission via organs transplanted from donors who had recently experienced homelessness. Those reports demonstrate the threat of severe bartonellosis in immunosuppressed organ transplant recipients after donor-derived *B. quintana* infection. Addressing the root causes of *B. quintana* transmission could improve the quality of life for persons experiencing homelessness and simultaneously mitigate risk for donor-derived *B. quintana* transmission. Interventions include improved access to housing, consistent access to hot water for showers and laundry, early treatment of body lice infestation and *B. quintana* infection, and *B. quintana* testing and prophylactic treatment of recipients of organs from donors who have experienced risk factors for *B. quintana*, including homelessness.

*Bartonella quintana* is a re-emerging, louseborne pathogen that can be insidious because of diverse clinical manifestations, laboratory diagnostic challenges, and disproportionate effects on marginalized populations that face substantial barriers to obtaining adequate healthcare. After *B. quintana* infection is diagnosed, treatment can be complex and costly, consisting of extended courses of antimicrobial drugs and sometimes requiring surgical intervention (e.g., cases of infective endocarditis). With increasing availability of molecular diagnostic tests that can identify *B. quintana*, outbreaks and infections have been detected with increasing frequency (*1*–*7*) among persons experiencing homelessness (PEH) and, more recently, among organ transplant recipients (*8*).

*B. quintana* is transmitted by human body lice (Figure 1), which can live in the clothing of persons who lack regular access to hygiene resources. In the United States, *B. quintana* infection occurs almost exclusively among PEH. Because the number of persons experiencing housing instability is increasing (*9*,*10*), the number of organ donors recently or currently experiencing homelessness at the time of death is also likely to rise (*11*). Housing status is not included in donor history questionnaires (*12*); thus, the frequency of homelessness among deceased organ donors is unknown. However, organ donors are often young persons who have experienced sudden and traumatic deaths; and because persons with housing instability experience disproportionate rates of physical trauma, opioid overdose, and early death (*13*), they may also be more likely than members of the general population to become organ donors.

**Figure 1:**
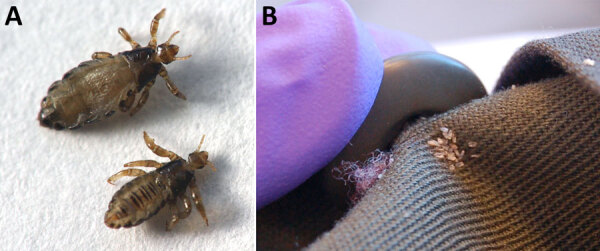
Dimorphous *Pediculus humanus humanus* (human body louse). A) The female adult (top) is larger than the male adult (bottom). B) Eggs, also known as nits, observed behind a coat button. Photographs courtesy of Denise Bonilla and the California Department of Public Health.

Beeson et al. describe an intensive investigation into *B. quintana* infections resulting from donor organ–derived transmission to 2 kidney recipients (*14*). In Canada, 6 confirmed cases of donor organ–derived transmission of *B. quintana* from donors with a history of experiencing homelessness were also recently reported (*8*). Those cases show that *B. quintana* transmission via organ donation can cause severe disease, probably because of the high level of immunocompromise resulting from medications designed to prevent organ rejection.

Opportunities for prevention and early diagnosis can be identified by analysis of homelessness epidemiology, body lice ecology, and *B. quintana* transmission dynamics. We closely examined those factors to determine specific strategies to reduce body louse infestation among PEH; promote early recognition, detection, and treatment of *B. quintana* infection among PEH; and mitigate transmission risk to organ recipients.

## Epidemiology of Homelessness in the United States

Since 2016, homelessness has increased in the United States, driven in part by increasing housing costs, the COVID-19 pandemic, and the opioid epidemic. The US Department of Housing and Urban Development (HUD) estimates that, on a single night in 2022, approximately 582,500 persons in the United States were experiencing homelessness; 40% of those were experiencing unsheltered homelessness in cars, tents, abandoned buildings, the street, or in other places not intended for human habitation (*10*). The HUD estimate of homelessness prevalence does not include persons with unstable housing who were able to stay with friends or relatives, sometimes called doubling up or couch-surfing, which has also been on the rise (*9*).

Homelessness is a nationwide issue; however, ≈20% of PEH in the United States live in New York, New York, or Los Angeles, California (*10*). The experience of homelessness differs by geographic area; sheltered homelessness is most common in New York, and unsheltered homelessness is more common in Los Angeles (*10*).

In the United States, PEH are most often cisgender men; rates of homelessness are almost twice as high among men than women (*9*). In 2022, approximately 1.1% of all PEH reported a gender other than cisgender (*10*,*15*); proportions among youth experiencing homelessness were higher (*15*). Rates of homelessness among many minority groups in the United States are disproportionate. The highest rates of homelessness among persons of any race/ethnicity are among persons who identify as Native Hawaiian/Pacific Islander (160/10,000 population), American Indian/Alaska Native (67/10,000 population), or Black/African American (55/10,000 population); nearly 40% of PEH identify as Black/African American (*9*). Hispanic/Latinx persons are also disproportionately affected, representing nearly one quarter of PEH in 2022, an increase from prior years (*10*). Barriers to adequate housing may be further amplified by intersectionality of those characteristics (e.g., when an individual’s race/ethnicity coincides with nonconforming gender or sexual orientation).

Homelessness is strongly associated with substance use (*16*). In the ongoing opioid epidemic, driven by readily available, highly potent synthetic opioids (e.g., fentanyl), drug overdose has become the leading cause of death among PEH (*17*). In Los Angeles, drug overdose accounted for 37% of all deaths among PEH in 2020 and 2021, outpacing other causes (e.g., coronary artery disease) (*18*).

Homelessness has well-documented adverse effects on health (*19*–*21*), resulting from structural and social barriers to healthcare, nutrition, and safe environments. Barriers are exacerbated by discrimination and stigma against PEH. Lack of consistent access to basic services, resources, and safety can result in inconsistent showering and laundering, which creates a risk for body lice infestation (*22*,*23*), which directly contributes to the greatly disproportionate rate of *B. quintana* infection and disease in PEH.

## Body Lice Ecology and Transmission Dynamics

Body lice (*Pediculus humanus humanus*), unlike the more common head lice, are not found directly attached to the human host; instead, body lice live on clothing, which provides easy proximity to human skin for frequent blood meals. Ideal conditions for body lice are 79%–90% humidity and 29°C–32°C (84.2°F –89.6°F) temperature, conditions that are often found in clothing next to human skin. Body lice cannot survive humidity <40% or temperatures >50°C (122°F) (*24*) and thus are easily killed when clothing is laundered in hot water (typically 60°C [160°F]). Adult body lice (Figure 1, panel A) live for weeks to months but can survive for only 2 days without a blood meal (*24*). They cement their eggs, also known as nits, to clothing fibers or seams (Figure 1, panel B), and the eggs hatch after 8–10 days (*24*). Body lice spread person to person through direct close contact or by shared infested clothing or bedding; dense living quarters with infrequent showering and clothes laundering present an ideal setting for body lice transmission.

Body lice are competent vectors of several bacterial pathogens, including *B. quintana*, *Rickettsia prowazekii* (the cause of epidemic typhus), and *Borrelia recurrentis* (the cause of louseborne relapsing fever). Humans are the primary reservoirs of *B. quintana*; a body louse becomes infected after taking a blood meal from a bacteremic human host. After infection, exponential replication occurs in the louse gut; up to 10^7^ *B. quintana* bacteria have been shown to be excreted in the feces of 1 louse in 1 day (*25*). An infected body louse remains infectious for its lifespan; louse feces can remain infectious for up to 1 year (*26*). When the infected body louse takes blood meals, which they do 1–5 times each day, they defecate and leave feces with high quantities of viable *B. quintana* near the bite site (*25*). A person becomes infected from inoculation of infectious louse feces through skin lesions or mucosal surfaces (*24*,*27*). The bite of a human body louse causes an intensely pruritic rash; scratching increases inoculation risk via the introduction of infected feces into abraded skin. *B. quintana* bacteremia in humans is often asymptomatic and chronic; durations of up to 8 years have been documented (*26*). Asymptomatic *B. quintana* bacteremia can result in further transmission to biting body lice and subsequent transmission to other persons, as well as increased risk of developing end-organ damage, including endocarditis (*28*).

Asking patients about itchy skin and bug bites can be helpful questions when screening for body lice infestation. Careful inspection of clothing may reveal visible body lice, nymphs, nits, or feces in the interior seams of clothing. A thorough dermatologic examination often reveals erythematous papules and papular urticaria, typically concentrated on the trunk where lice-infested clothing is in direct contact with the skin. Because of the severe pruritis, extensive excoriations are common and severe, and chronic infestation can lead to postinflammatory increased pigmentation and skin thickening (*24*). In addition to bacterial infections transmitted by body lice, severe infestation can also result in iron deficiency anemia and eosinophilia (*24*,*29*).

Body lice infestations occur among persons in crowded living situations who lack access to hygiene resources. PEH often lack consistent access to hot water for bathing and laundry, which are the primary tools for preventing and treating body lice infestation (*30*–*32*). Homeless shelters are often crowded, increasing risk for close person-to-person contact, which promotes body lice transmission. Some PEH may receive donated clothing and bedding, which are not always washed with hot water before distribution (*31*). Sharing of resources such as clothing and bedding by PEH further increases risk for spread of body lice. Mental health and substance use conditions may also be barriers to accessing laundry and bathing facilities, even when they are available (*33*). Additional barriers may be other social factors (e.g., risk for personal violence or theft of possessions while accessing bathing or laundry facilities). Combined, those factors result in inadequate personal hygiene practices among PEH, perpetuating disproportionate lice infestation and louseborne disease. One study in San Francisco, California, reported finding body lice infestations in nearly 30% of PEH (*30*). Another study in Marseille, France, found that body lice infestation was associated with 2.75 (95% CI 1.14–6.65) increased odds for *B. quintana* bacteremia (*34*).

## *B. quintana* Epidemiology and Clinical Overview

Molecular evidence indicates that *B. quintana* has infected humans for >4,000 years (*35*). First recognized around 1914 during World War I, *B. quintana* infection was primarily known as trench fever or quintan (5-day) fever, an acute syndrome of fever lasting 1–5 days and recurring at 5-day intervals, accompanied by shin pain, fatigue, headache, and splenomegaly. During World War I, trench fever was estimated to have affected more than 1 million troops (*36*). For most of the 20th century, trench fever was considered a disease of wartime or humanitarian crises, and outbreaks occurred again during World War II (*22**,**28*). The body louse was identified as the vector of *B. quintana* in the mid-1920s (*22*), but it was not until 1961 that the gram-negative bacterium was successfully isolated in culture (*22**,**28*). Since its initial recognition, the bacterium has been reclassified several times; initially known as *Rickettsia quintana*, its name was later changed to *Rochalimaea quintana* and then, in 1993, to the current *Bartonella quintana.*

Until the 1980s, *B. quintana* was only known to cause trench fever, the acute symptomatic disease with a relapsing fever pattern, the likely result of an intraerythrocytic phase of infection followed by periodic erythrocyte rupture (*27**,**37*). In the early years of the HIV/AIDS epidemic, however, *B. quintana* and the closely related species *B. henselae* were identified as the cause of bacillary angiomatosis, causing extremely vascular lesions of the skin. In addition, *B. henselae* was identified as the sole species causing peliosis hepatis, resulting in highly vascular lesions in the liver. Both of those novel vascular proliferative lesions occurred among persons living with advanced immunosuppression caused by AIDS (*38*). In the 1990s, a diverse array of clinical manifestations from *B. quintana* infection were identified, including subacute endocarditis and chronic bacteremia, which disproportionately occurred among PEH who did not have known HIV infection or other immunodeficiency (*39**,**40*). The prevalence of asymptomatic bacteremia is unknown because of limitations of diagnosis and underreporting, but bacteremia durations of months to years have been described (*26*).

Because *B. quintana* infection is not a nationally reportable condition, most of what is known about its current epidemiology is based on seroprevalence studies. US studies conducted since 1996 have reported *B. quintana* seropositivity rates among PEH of 5%–15% (*1*,*2*,*41*). In recent years, outbreaks and *B. quintana* seropositivity have been documented among PEH in geographically diverse urban areas of the United States, including Denver, Colorado (*1*,*3*); Anchorage, Alaska (*4*); Seattle, Washington (*2*); San Francisco, California (*6*); Baltimore, Maryland (*7*); New York, New York (*42*); and Washington, DC (*5*). Cases have also been reported in Canada (*43*). Independent risk factors for *B. quintana* seropositivity identified among PEH include duration of homelessness >1 year, age >40 years, and alcohol use disorder (*44*). Intravenous drug use has been identified as another potential risk factor; *B. quintana* seropositivity among persons who use intravenous drugs, irrespective of homelessness, has been reported as 2%–10% in the United States (*7*,*45*). However, a notable limitation of those serologic studies is the potential for cross-reactivity with antibodies to other bacteria, including other *Bartonella* spp., *Chlamydia* spp., and *Coxiella burnetii* (*46*).

*B. quintana* is challenging to grow in bacterial culture because it is slow growing (doubling time is ≈21 hours) (*25*) and requires special conditions. When *B. quintana* is suspected, cultures should be held for a minimum of 14 days, much longer than the typical 5-day incubation period for bacterial cultures (*3*). Until recently, serologic assays provided the primary diagnostic approach for detection of *B. quintana* infection, despite challenges with specificity. The most common serologic assay, an indirect immunofluorescent antibody assay, is also limited by low throughput. Although PCR diagnosis was described as early as 1994, molecular diagnosis for detection of *B. quintana* has only recently become more widely available for clinical diagnosis (*39**,**47*). In a 2023 study describing 430 cases of *Bartonella* spp. infection diagnosed by molecular methods, 15% were positive for *B. quintana*; of those, 82% were in male patients (compared with 18% in female patients), and 83% were in persons 18–65 years of age (*39*). Cell-free DNA testing has also recently emerged as a promising diagnostic tool for detecting *B. quintana* (*48*).

After *B. quintana* infection is known or suspected, treatment typically consists of doxycycline or macrolide antimicrobial therapy for 4–6 weeks, although high-quality evidence for the optimal antimicrobial regimen and duration is still needed. Effective treatment of endocarditis and bacteremia may require months of multiple antimicrobial drugs and should include doxycycline or a macrolide (both bacteriostatic) with the addition of a bactericidal drug such as rifampin (*49**,**50*). Surgical cardiac valve replacement is often necessary for cases of endocarditis (*51*), which often affects normal valves. Definitive treatment also requires controlling any ongoing body lice infestation to prevent reinfection and further transmission.

## *B. quintana* Prevention, Screening, and Early Treatment

**Figure 2:**
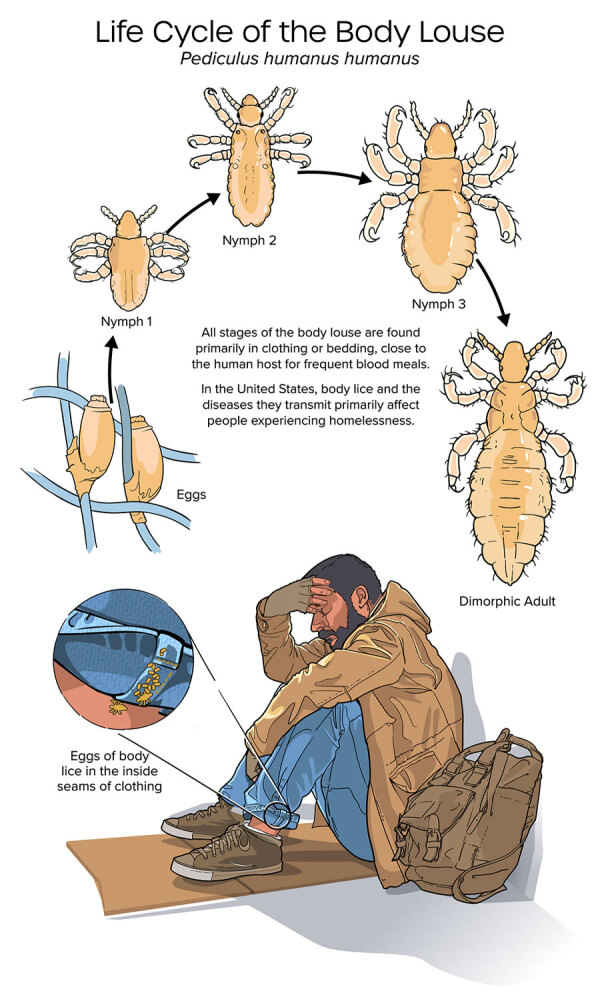
Life cycle of the human body louse (*Pediculus humanus humanus*).

Body lice infestation and *B. quintana* infection and disease can be prevented by promoting universal housing, increasing access to showers and laundry with hot water, and identifying patients at risk for infection so they can receive early diagnosis and treatment (Figure 2). Public health and clinical professionals have an opportunity to implement those interventions for PEH, the group at highest risk for disease and often from marginalized and minority groups, as well as for organ transplant recipients, who are often uniquely vulnerable to severe disease because of immunosuppressive treatment.

An ethical discussion of whether PEH should be organ donors is ongoing; by current transplant protocols, PEH are usually not considered for receipt of transplanted organs (*11*). Although the proportion of organ donors with a history of homelessness is unknown, PEH currently donate organs for transplantation in the United States; thus, risk for *B. quintana* transmission through organ transplantation should be recognized and mitigated.

**Figure 3:**
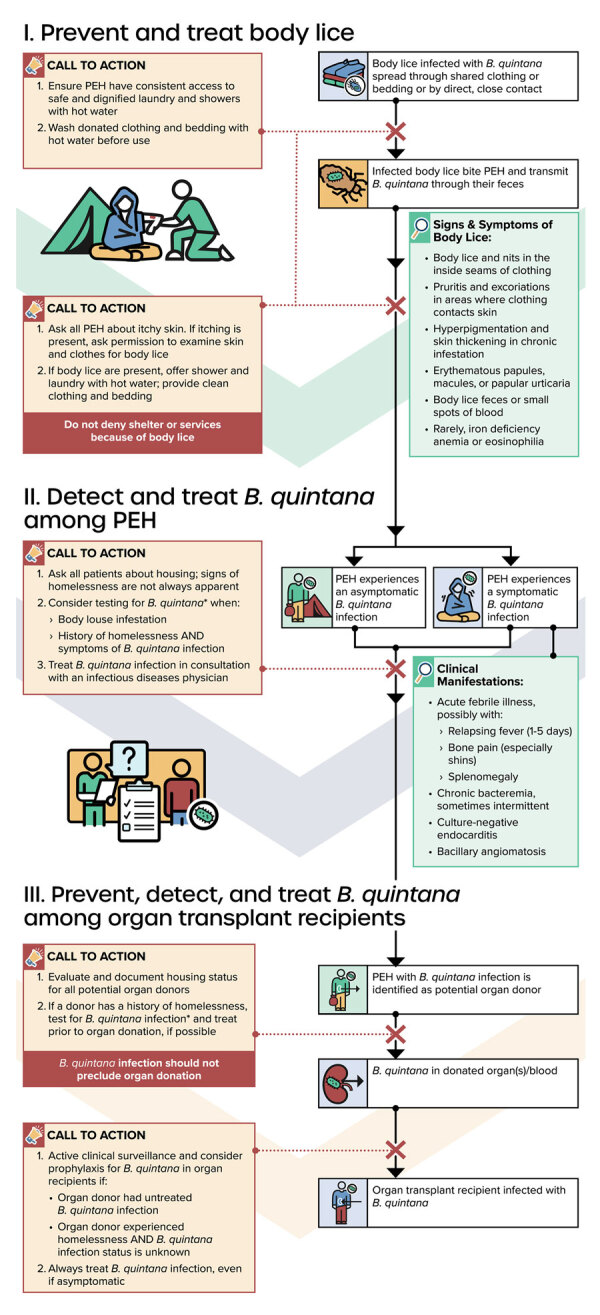
Conceptual framework for reducing transmission of *Bartonella quintana* in the United States among PEH and among organ transplant recipients through universal access to hygiene services, prevention and treatment of body lice infestation, and early diagnosis and treatment of *B. quintana* infection. Diagnostic testing for *B. quintana* includes bacterial culture with prolonged incubation time (minimum 14 days), serology, and molecular diagnostic methods (e.g., PCR or microbial cell-free DNA testing). PEH, persons experiencing homelessness.

With the guiding principles of primary prevention, health equity, and risk mitigation, we make the following calls to action (Figure 3):

- Homeless service systems
- Ensure that PEH have consistent, low-barrier access to basic hygiene services, including laundry and showers with hot water. Hygiene services should be offered with dignity and respect while addressing potential challenges of mental health conditions, substance use, risk for sexual violence, and the risk of losing one’s belongings or shelter.
- Launder donated clothing and bedding with hot water before distribution to PEH.
- For PEH with body lice infestation, offer a hot shower and a clean change of clothing and bedding. Exercise contact precautions when handling used clothing or bedding to limit infection transmission risk. In general, shelter, resources, and services should not be withheld because of body lice infestation, which is both preventable and treatable.
- Consider referring clients with body louse infestation to health services for *B. quintana* screening and facilitate access to treatment, if possible.
- Clinicians
- Ask all patients about current and previous housing status. Housing status should be systematically entered into medical records.
- Evaluate PEH for body lice infestation in a respectful way by asking a screening question about itchy skin and performing a physical examination. If itching is reported or if excoriations are observed, obtain consent to examine clothing for evidence of body lice. Exercise contact precautions when examining patients with lice infestation to limit infection transmission risk. Treat body lice infestations promptly by coordinating access to a hot shower and a clean change of clothing/bedding.
- Consider testing for *B. quintana* in patients who have evidence of body lice infestation or a history of homelessness and symptoms compatible with *B. quintana* infection. Note: Diagnostic testing for *B. quintana* includes bacterial culture with prolonged incubation time (minimum 14 days), serology, and molecular diagnostic methods (e.g., PCR or microbial cell-free DNA testing).
- Consider empiric treatment or prophylaxis for *B. quintana* for recipients of organs donated by persons with untreated *B. quintana* infection or by persons with a history of homelessness and unknown *B. quintana* infection status.
- Treat all patients with *B. quintana* infection, even if asymptomatic, in consultation with an infectious disease physician.
- Organ donor organizations
- Ask all potential organ donors and donor next of kin (for deceased donors) about current and previous housing status in a respectful and dignified way. Housing status should be systematically entered into medical records and reported by regional transplantation organizations.
- Consider screening all organ donors with a known history of homelessness for *B. quintana* and report test result to appropriate public health and medical organizations. Note: Diagnostic testing for *B. quintana* includes bacterial culture with prolonged incubation time (minimum 14 days), serology, and molecular diagnostic methods (e.g., PCR or microbial cell-free DNA testing).

With implementation of the strategies outlined, rates of body lice infestation and bartonellosis from *B. quintana* infection would decrease among PEH and among organ transplant recipients. A future without *B. quintana* infection is achievable if a cohesive, comprehensive approach is adopted that prioritizes universal access to basic hygiene resources.
